# Impact of cocreation training capsules for preschool teachers on children’s healthy habits: a pilot study conducted in Barcelona, Spain

**DOI:** 10.1186/s12889-021-12160-2

**Published:** 2021-11-14

**Authors:** Verónica Violant-Holz, Carlota Rodríguez-Silva, María Carol, Manuel J. Rodríguez

**Affiliations:** 1 Department of Didactic and Educational Organization, Faculty of Education, Universitat de Barcelona, Barcelona, Spain; 2 International Observatory in Hospital Pedagogy, Universitat de Barcelona, Barcelona, Spain; 3 Institute for Lifelong Learning Foundation (IL3-UB), Universitat de Barcelona, Barcelona, Spain; 4 Current address: Academic Area, Universitat Politècnica de Catalunya, Barcelona, Spain; 5 Department Biomedical Sciences, Institute of Neurosciences, Faculty of Medicine and Health Sciences, Universitat de Barcelona, c/Casanova 143, E-08036 Barcelona, Spain; 6 August Pi i Sunyer Biomedical Research Institute (IDIBAPS), Barcelona, Spain; 7 Networked Biomedical Research Centre for Neurodegenerative Disorders (CIBERNED), Barcelona, Spain

**Keywords:** Cocreation design, Health education, Healthy people, Preschool children, Health prevention, All@once program

## Abstract

**Background:**

Healthy habits are essential for preschoolers to have a healthy lifestyle. The promotion of these healthy habits from a holistic approach by preschool teachers guarantees a better quality of life and a healthier society. Using cocreation, we designed training for healthy habit promotion for preschool teachers (all@once). Then, we implemented the training and evaluated its impact on classroom teaching strategies.

**Methods:**

This study presents the all@once training design and its implementation and evaluation during 2019. The cocreation process involved 8 parents, 9 preschool teachers and 9 health professionals (selected by a nonprobabilistic sampling system according to quotas) to design training from a holistic perspective. To evaluate the all@once impact in classroom practice, a pilot study was undertaken in four public schools in Barcelona (Spain). All@once was implemented with 16 volunteer teachers selected by convenience sampling and 328 children. A mixed methods approach was chosen to collect data based on direct nonparticipating naturalist systematic observations in June and October 2019. After qualitative data categorization, changes in health routines and actions at school were assessed by either contingency table analysis of frequency distributions or nonparametric comparisons of two related samples.

**Results:**

The cocreation process provided training organized into online capsules with a holistic view of health in four main dimensions (nutrition, hygiene, physical activity and emotional health). Of these dimensions, the emotional health dimension comprised half of the training content. Pilot testing of the impact of all@once on classroom health-related activities evidenced an increase in the likelihood of observing fruit consumption by children, healthy habit promotion and hand washing. The most significant all@once-induced changes that we observed were related to teaching strategies concerning the emotional health dimension of the training.

**Conclusions:**

This pilot study provides evidence of cocreation being a productive way to design training for preschool teachers regarding inclusive education in integral health. This approach collects the needs of the school community, provides training with a holistic concept of health and effectively impacts classroom routines and family health habits in the short term.

## Background

The concept of health currently focuses on the ability of people to adapt and self-manage in the face of social, physical, and emotional challenges [1]. This modern concept proposes six dimensions of health, namely, bodily functions, mental functions and perceptions, spiritual dimension, quality of life, social and societal participation, and daily functioning [2]. These dimensions are related to three domains, namely, social, mental, and physical health, and they include the characteristics of wellbeing and resilience [3]. All of these aspects of health can be promoted at early ages, in line with the Sustainable Development Goals (SDGs) for 2030 [4] and the Whole School, Whole Community, Whole Child (WSCC) model [5]. This notion of health encompasses metabolic, cardiovascular, infectious, and psychiatric diseases, among others.

A growing health problem in Western societies is the increasing prevalence of overweight and obesity, especially in childhood, when these factors are considered predictors of adult obesity and have consequences for both the individual and society as a whole [6]. Childhood represents a critical period for interventions because healthy lifestyle habits develop during this period, especially during the first years of life [7, 8]. There are recommendations for targeting not only overweight or obese children but also healthy children, teenagers and adults of all ages. Thus, school-based interventions have been considered useful and pertinent for this issue by addressing healthy eating, increasing physical activity, and improving body image [9].

Thus, schools emerge, alongside families, as the major field where children learn about health and lifestyle habits because teachers and parents are both central figures for role modeling healthy behavior [10, 11]. It is therefore meaningful to study the perceptions of teachers and parents to improve their knowledge and training [12–18] and to develop interventions as part of the fundamental processes for health promotion and education [19, 20].

To our knowledge, the existing interventions involving the school community focus on only one or two dimensions of health [21–25]. Other interventions specifically address children with overweight and obesity [26] without considering children’s health from a holistic perspective. Thus, some reported interventions targeting preschoolers by the whole school community improve health regarding nutrition and feeding [27, 28], physical activity [29, 30], hygiene [31, 32], or emotional health [33, 34]. However, all of them are designed as a single dimension of intervention. Physical educators and health educators thus have the opportunity to collaborate with each other and promote the development of positive decision-making skills for healthy behaviors [35]. There is also evidence of the effectiveness of online training for preschool teachers regarding physical activity. These studies report that online training allows for great dissemination, provides public health impacts and creates lifestyle changes in preschoolers [36–39].

Studies have suggested that interventions are more meaningful and sustainable if they come from the communities for which they are designed via the involvement of all the stakeholders in a cocreation process [40]. This pilot study is thus a contribution to this line of research. It responds to the need to adapt trainings for teachers designed in cocreation from holistic thinking by listening to the voices of teachers, parents and health professionals. Our cocreation design involves training users (preschool teachers) and those who impact children’s healthy habits (parents and health professionals), thereby taking all part in the whole design process. There is evidence that cocreated public health interventions are a feasible design for reducing sedentary behavior in adulthood [41]. Such programs also allow caregivers to recognize themselves as cocreators of healthful eating behavior among their young children, both in the short and long term [42].

### Aim of the pilot study

In this study, we analyze the impact of cocreated training for preschool teachers in classroom routines and children’s healthy habits in the short term. To approach this objective, we designed and tested all@once online training for preschool teachers to promote young children’s health from a holistic perspective. The training was developed with stakeholders through cocreation sessions. The all@once training was then implemented, and its impact on short-term outcomes was evaluated in four educational centers in Barcelona, Spain. The cocreation process provided online training with a holistic perspective of health. All@once implementation and assessment in this pilot study resulted in discrete changes in classroom routines and family health habits in the short term.

## Methods

### Mixed methods research design of the pilot study

**Fig. 1:**
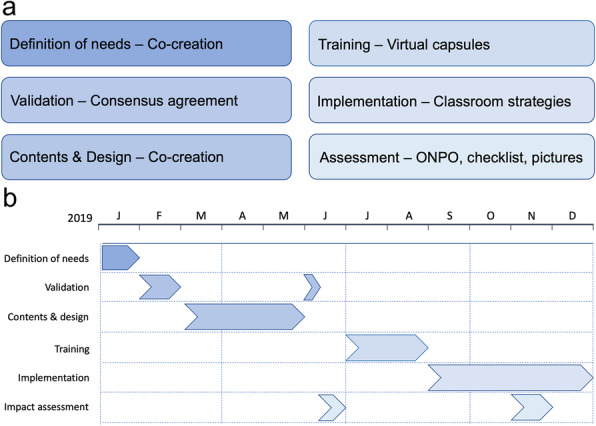
Methodological research design. (a) Graphical panel summarizing the methodological phases and instruments used. In the phase of training content & design, cocreation included 4 stages: identification of needs, categorization: prioritization and validation. ONPO: overt nonparticipating naturalist systematic observation. (b) Gantt chart illustrating all@once project schedule

In the current pilot study, we used a mixed methods research design that combined observational and selective methodologies in each phase of the study, following the Guidelines for Reporting Evaluations based on Observational Methodology (GREOM) [43]. Our research design followed an observational methodology that was carried out with both direct observation (data grid) and indirect observation (picture collection with checklist) [44]. These qualitative data were then analyzed and integrated into quantitative data [45]. This approach made it possible not only to materialize the complementarity between quantitative and qualitative methods but also to involve them throughout the research process: data, design, analysis, interpretation, and paradigms [46–48]. To triangulate the quantitative and qualitative data, the four phases related to the field format [49] were followed, namely, establishment of the criteria (dimensions and response levels), elaboration of a catalog for each criterion (categories and subcategories), assignment of a decimal coding system (alphanumeric assignment for each observed behavior with a hierarchical system) and elaboration of the coding list (which allowed the combination of the field format combined with the category system, and consequently the preparation of the qualitative data to be treated as quantitative data). Figure 1 summarizes the methodological phases of the pilot study process.

We designed all@once with a cocreation method involving 26 participants. They were recruited by a nonprobabilistic sampling system according to quotas. Our training was implemented in four public schools in Barcelona (Spain) and included 16 teachers selected by convenience sampling and 328 children. To evaluate the impact of all@once in classroom practices, a mixed methods approach was chosen, and before-and-after evaluation was performed. Data collection was based on overt direct nonparticipating naturalist systematic observation (ONPO) that included a data grid and overt indirect ONPO with picture collection of teachers and preschoolers’ behavior. ONPO was performed in June and October 2019. After qualitative data categorization, changes in health routines and actions at school were assessed by either a contingency table analysis of the frequency distributions or nonparametric comparisons of two related samples.

### Cocreation design, setting and procedure of the all@once training

A qualitative research method was chosen to design the all@once training program. The training design was participatory and based on a cocreation method defined by Mukhtar et al. [50]. This method considers customers or end users to be experts who take part in product design. In our approach, preschool teachers, families, and health professionals were considered cocreators in the training design [51]. Three cocreation sessions were held in Barcelona (Spain) in a neutral place for families, teachers, and health professionals outside of their immediate environment and always in the same classroom [52]. The sessions were organized by researcher MC and conducted by researcher VV-H. The duration of each session was limited to less than 3 h.

Cocreation sessions were developed in four stages [53]. First, training needs were identified using the following question: “What should teachers know to improve children’s health in general?” to induce brainstorming. Participants identified needs individually and then worked in pairs or trios. Second, for the production of ideas and categorization, participants shared and generated ideas for categorization in thematic groups. Third, participants prioritized the categorized thematic groups following the diamond technique, where in consensual criteria [54, 55] among the participants, the contents that the group considered relevant and necessary were prioritized [56]. Fourth, in the validation phase, 12 experts in the field analyzed the three diamonds and organized the all@once training accordingly using the consensus agreement method [54, 55].

### Cocreation participants, recruitment process and eligibility criteria

Participants in the cocreation sessions came from three different population groups: parents, teachers and health professionals. For the enrollment of parents and teachers, schools in Barcelona (Spain) were approached in collaboration with the *Consorci d’Educació de Barcelona,* which is the main educational authority for the city’s primary and secondary education establishments’ organization. Schools were selected by nonprobabilistic convenience sampling according to their availability and willingness to participate in the study and their allocation in different city neighborhoods, thus guaranteeing the inclusion of participants from different sociocultural strata. In the first stage, a call for interest was launched with positive answers from several schools. Then, personalized assessments through e-mail and life interviews were scheduled to duly explain the study. Therefore, engagement was enhanced by voluntary participation in the project regarding the following aspects: parents of 3- to 6-year-old children attending the schools participating in the project had to commit to attending several meetings; teachers had to be fluent in English (as some of the training contents were developed in this language), specialists in early childhood education (preschool teachers for children aged 3 to 6 years) had to commit to attending several meetings and go throughout a pilot training course; and finally, these individuals had to be able to transfer information to their colleagues and to the director of the center upon request. In addition, health professionals were enrolled, including practitioners, nurses, pediatricians, psychologists and other medical staff of the pediatric *Hospital Sant Joan de Deu* in Barcelona (Spain)*.* A total of 26 people (17 female and 9 male) participated in the three cocreation sessions in the three different groups mentioned above: 8 parents, 9 preschool teachers, and 9 health professionals. The sampling system was nonprobabilistic according to quotas, where each group (families, teachers, and health professionals) was seen as a sampling unit. Their work ran in parallel, and their outputs and answers were not transferred from one group to another until the very end of the process; even at this point, transference was only done for information purposes by gathering all data and thus following strict anonymization procedures. The criterion of the maximum variability of participants and contexts was sought [57]. Additionally, we aimed for a gender balance in all three groups, and the final enrollment of the participants was voluntary once basic requirements (such as timings and commitment) were fulfilled. Finally, a total of 12 experts in related fields (biochemistry of nutrition, education management, medicine, nursing, pedagogy, pediatric psychology, and sport physiology) from four European countries (Denmark, France, Netherlands, and Spain) participated in the validation phase of the cocreation session.

### Assessment design, setting, procedure of all@once training implementation, and impact assessment

An evaluation before and after the training implementation was conducted to assess the influence of the all@once program on teaching strategies in the daily practices of the preschools. The evaluation followed a single session observational method study [43] and had a diachronic cohort design. A mixed methods approach was used. For the objective qualitative analysis of school routines, observation was performed using direct ONPO [58] to capture the behaviors in the classroom, with a data grid that implied total perceptibility. Moreover, indirect ONPO was performed to capture pictures in different spaces of the school [59], with a checklist that implied partial perceptibility.

The evaluation dimensions and categories were constructed from the theoretical content of the all@once training program developed in the cocreation sessions. Once the instruments were constructed, an initial evaluation was carried out in the classrooms. Subsequent validation by consensus [54] between two researchers allowed the adjustment of the instruments before returning to the classrooms. Only common elements from the initial and final evaluations were used for data analysis.

### Assessment participants, recruitment process and eligibility criteria

For implementation of the all@once training, we performed a preliminary pilot study with an intrasessional diachronic analysis [43] where data were collected before and after the training attendance. Teachers were selected from four public schools in Barcelona (Spain), as mentioned above. Educators were recruited on a voluntary basis provided they had successfully fulfilled all four educational modules of the all@once training. It was indeed a peer-to-peer approach based on trust and the common understanding between the parts; researchers would observe the children’s behaviors and reactions while preventing them from interfering in the development of the class. Consequently, children were approached by their teachers, who remained the conductors of the classwork. It was through the teacher’s authority and collaboration that researchers came inside the classroom to perform their nonintrusive observations.

Furthermore, the parents of children in a classroom where the teacher joined the study signed an informed consent form. Aligned with previous rationale, children whose guardians refused to join the study and/or would not fulfil the above listed requirements were automatically excluded from the study itself. The researchers duly noted such situations. However, only one class was excluded because the teacher could not ultimately join the study for personal-professional reasons. Because the voluntary approach was taken, researchers ensured the full engagement of the teachers participating in the process. Ultimately, 16 teachers (12 female and 4 male) and 328 children (99 children, 3–4 years old; 102 children, 4–5 years old; and 127 children, 5–6 years old) participated in the assessment and were selected by nonprobabilistic convenience sampling. Some of the teachers also participated in the cocreation sessions.

### Data collection and analysis regarding the all@once training assessment

Seven observers collected the direct and indirect ONPO data, including taking pictures of classroom activities. Two of these observers designed the instruments and trained the others in the assessment method and instrument use in two sessions. During the actual data collection, a coordinator provided feedback on data quality and assessment criteria [52]. To analyze the impact of the all@once training on the work of teachers, direct ONPO was performed in two sessions. The first session was performed before teachers attended the training and aimed to establish their background activities related to the training objectives. This session also made it possible to refine and adapt the evaluation instruments according to the cohort characteristics. A second assessment session was performed after the teachers attended the training. Data collection was thus conducted at each school in June and October 2019, with two observations per session. In both sessions, all schools were visited for assessment during the same week. All data collection sessions were performed from 9:00 a.m. to 13:30 p.m. Teachers and children knew in advance the dates and times when observers would be present to collect data. Observers stood at the back of the classroom, thereby minimizing their interactions with teachers or children.

Data grid and checklist assessment instruments were raised and constructed from the prioritization and validation phases of the cocreation sessions, which elicited 128 qualitative items categorized in all four health dimensions of the training. For each dimension, the 128 items were regrouped into 8 categories for the dimension of nutrition, 5 categories for the dimension of physical activity, 6 categories for the dimension of hygiene, and 5 categories for the dimension of emotional health. Once the first ad hoc version of the instrument had been constructed by two of the team’s researchers, a consensus meeting [55] was held among the rest of the research team in charge of making the observations. The instrument was refined by consensus [55] with the first staging.

According to their relevance for the evaluation, these items were unified into 24 subcategories, following the categorization system that was constructed during the validation phase of the cocreation session. The total number of subcategories was as follows: 8 for nutrition, 5 for physical activity and body awareness, 6 for hygiene, and 5 for emotional health. Data from these subcategories were graded into two values—observed vs. nonobserved—using consensus agreement between two researchers. Differences in the frequency distribution between the first (June) and second (October) assessments were estimated in 2 × 2 contingency tables. For each subcategory, odds ratios (ORs) with 95% confidence intervals (CIs) were calculated to quantify the all@once effect in the paired binomial proportions; the McNemar mid-p test was performed to assess the significance of the effect [60, 61].

Data analysis from pictures was conducted following the same procedure as that used for the data grid and checklist. Qualitative data were thus categorized using consensus agreement between the two researchers. Pictures were reviewed one by one and classified into the dimensions named in the all@once training, according to which each training objective was met by the classroom activity shown. As pictures are a multidimensional instrument [62] and may reflect more than one evaluated item, some pictures were included in more than one dimension. Differences in the number of classroom activities observed between the first (June) and second (October) assessments were analyzed by the nonparametric paired Wilcoxon signed-rank test. Data are presented as the mean ± standard error of the mean (SEM). In all statistical analyses in this study, differences were considered significant with *p* values < 0.05. Analyses were performed with the SPSS Statistics v26 (IBM Corp., USA) statistical package.

## Results

### Teacher training needs and all@once content design

**Fig. 2:**
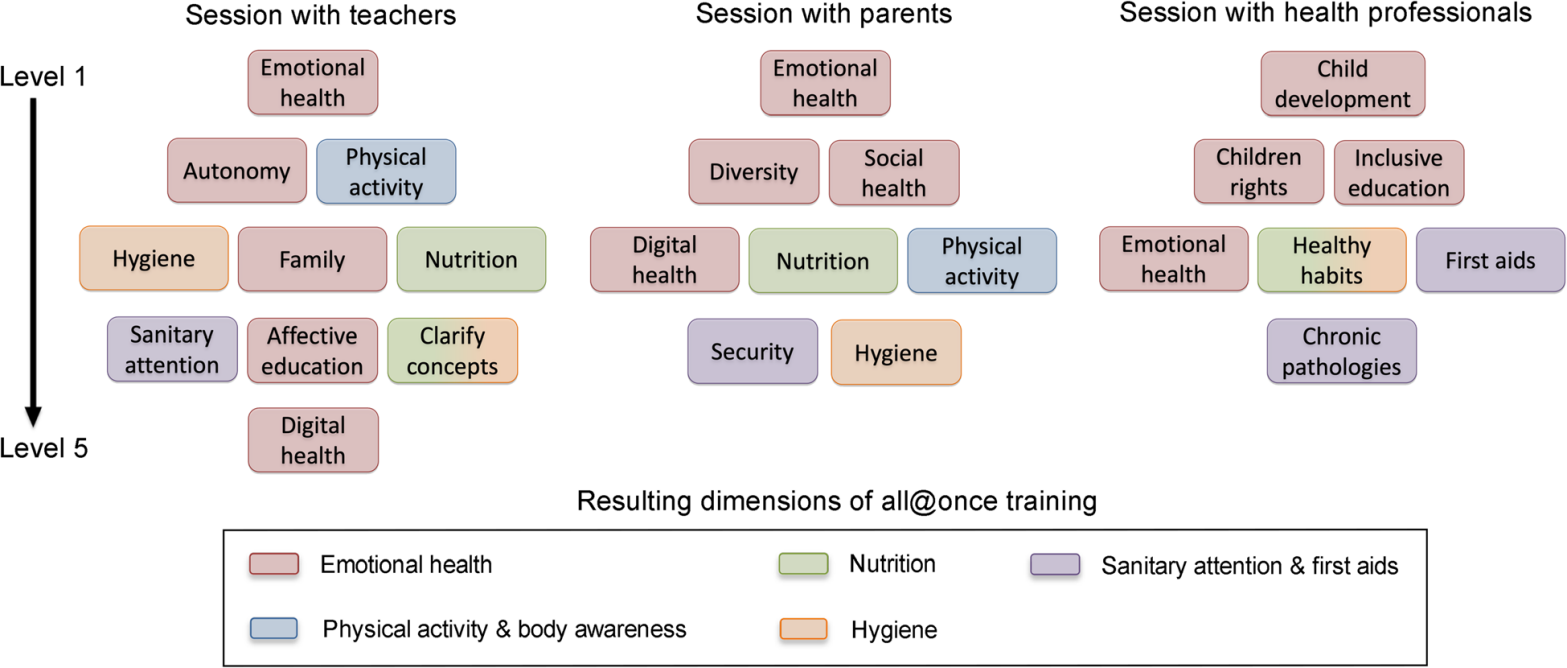
Organization of all@once training content generated in the cocreation process. Charts show the diamonds resulting from cocreation sessions with stakeholders; these categorize and prioritize the raised health training needs, shown as items labeled in colored squares. The bottom square shows the final five dimensions defined in the validation phase. The color of the diamonds represents the dimension in which the item was included in the validation phase. The so-called emotional health dimension includes emotional, social, and psychological and motor developmental aspects (see text for details)

Cocreation sessions with parents, teachers and health professionals to define, categorize, and prioritize the training needs of teachers to improve children’s health reached similar results. All three groups prioritized those needs that focused on children’s psychological health and development (Fig. 2). The cocreation session with teachers identified a total of 31 needs to improve their health competence. Teachers categorized these needs into 10 groups and prioritized the category they called emotional health as their most important need, followed by the items of autonomy and physical activity, while the hygiene and nutrition items only appeared at the third prioritization level (Fig. 2). The cocreation session with families identified a total of 26 needs for teachers. They categorized these needs into 8 groups and prioritized the category of emotional health, while those of nutrition, physical activity, and hygiene appeared at the lowest prioritization levels (Fig. 2). The cocreation session with health professionals identified a total of 28 needs for teachers. They categorized these needs into 7 groups and prioritized the item of child development as the most important; in the group called healthy habits, they included all aspects related to nutrition and hygiene. This item was classified at the third level of the prioritization diamond, just above items on sanitary knowledge, including first aid and chronic pathologies. This group did not identify any teacher training needs regarding physical activity (Fig. 2).

During the validation process performed by consensus agreement, 12 experts in the field from four countries analyzed the cocreation result diamonds and organized the all@once training. They fit all of the training contents into five final dimensions of the course: a) nutrition, with the 5 subdimensions of healthy lifestyle, water intake, palate education, the social dimension of eating, and healthy habits in the family; b) physical activity and body awareness, with the 5 subdimensions of the psychomotricity class, playtime on the patio, sedentarism reduction, movement in daily routines, and physical activity outside the school; c) hygiene, with the 5 subdimensions of dental hygiene, body hygiene, hand washing, hygiene of spaces, and hygiene of the posture; d) emotional health, with the 5 subdimensions of my own feelings and emotions, the feelings and emotions of the others, adaptation and regulation, emotional development, and challenges and setbacks; and finally, e) sanitary attention and first aid (Fig. 2).

These experts also defined the training content following the guidelines of the cocreation diamonds; therefore, the emotional health dimension, which was thought to be an integral approach to cognitive development, physical development, and different stages of development in the child, comprised half of the training time. The sanitary attention and first aid dimension was classified as a complementary dimension. The final training instrument was designed as online capsules organized around the four main dimensions of the training, which were presented with a holistic view of health. To attend to the complementary dimension, one of the capsules included a list of resources with information on sanitary attention and first aid.

### Impact of all@once training on school health-related activities

**Fig. 3:**
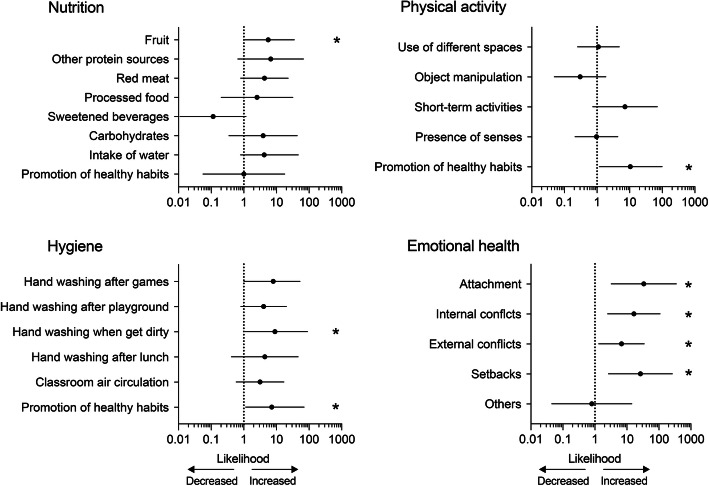
Impact of all@once training for healthy habits. Graphs show the odds ratio with 95% CI for assessed items as a quantification of the likelihood of being observed after the teachers’ attendance at training. *, *p* < 0.05, McNemar mid-p (*n* = 16 teachers and 328 children per session)

The attendance of teachers in the all@once training induced discrete changes in the everyday activities and routines at schools and in the healthy habits of children. Eight out of the 24 analyzed subcategories showed statistically significant changes in paired binomial proportions between June and October (Fig. 3). In the nutrition dimension, teachers’ attendance at the training was slightly associated with changes in the children’s mid-morning lunch. The likelihood of bringing fruit from home significantly increased in October with respect to June (OR = 5.570, 95% CI: 1.088–35.268; McNemar mid-*p* = 0.032). We found no significant changes in the other parameters assessed, including the likelihood of observed water intake by children and the promotion of healthy eating habits among school staff. In the physical activity and body awareness dimension, we only found an association between the training and an increase in the probability of the promotion of healthy physical activity habits (OR = 10.500, 95% CI: 1.176–98.478; McNemar mid-*p* = 0.032; Fig. 3). Similarly, in the hygiene dimension, all@once training significantly increased the likelihood of children washing their hands after getting dirty (OR = 9.000, 95% CI: 1.100–89.612; McNemar mid-p = 0.032) and in the promotion of healthy habits by the schools’ personnel (OR = 7.200, 95% CI: 1.138–70.200; McNemar mid-p = 0.032; Fig. 3).

Comparisons of data from the emotional health dimension revealed differences in paired binomial proportions between June and October in 4 out of the 5 subcategories analyzed (Fig. 3). We found a significant increase in the likelihood of observing the resolution of children’s attachment situations (OR = 33.750, 95% CI: 3.245–351.052; McNemar mid-*p* = 0.011), internal conflicts (OR = 16.500, 95% CI: 2.507–108.595; McNemar mid-*p* = 0.021), external conflicts (OR = 6.750, 95% CI: 1.318–34.565; McNemar mid-*p* = 0.032), and setbacks (OR = 26.400, 95% CI: 2.653–262.690; McNemar mid-*p* = 0.018).

**Fig. 4:**
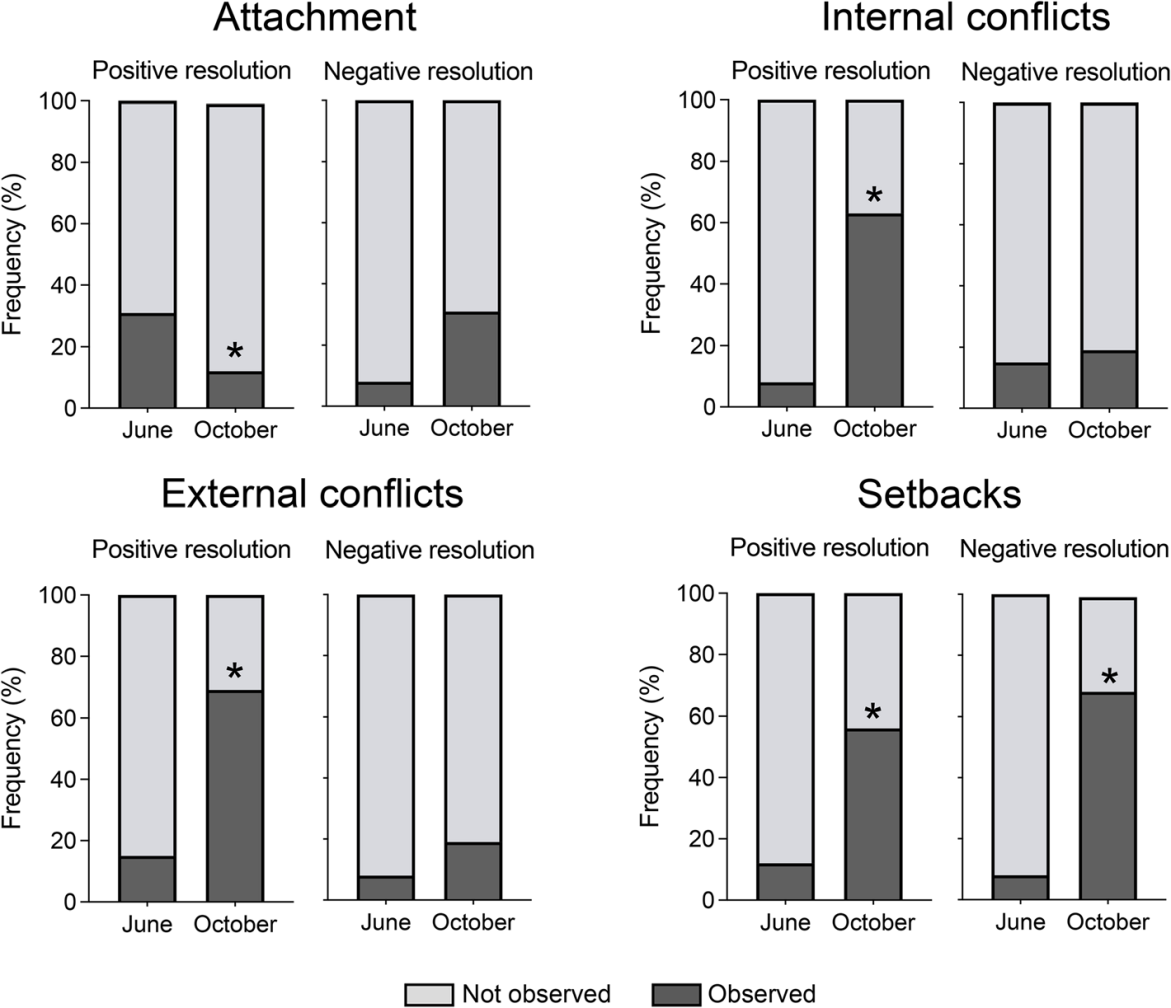
Effects of all@once training for emotional health. Histograms show the relative frequencies of either positive or negative resolution strategies displayed for teachers in the classroom before (June) and after (October) training attendance. *, *p* < 0.05, McNemar mid-p, different from June. (*n* = 16 teachers and 328 children per session)

Teacher interventions for resolving situations related to emotional education observed among children were then classified according to their adjustment to all@once learning outcomes. Interventions were thus classified as positive resolutions (teacher interventions that facilitate understanding of emotions) or negative resolutions (teacher interventions that do not take into account that emotions can be understood). A detailed analysis of these teachers’ strategies showed no clear effects of the training (Fig. 4). Comparisons of the strategies in emotional education between June and October showed that the frequency of strategies for positive resolution to solve attachment moments decreased significantly in October (67% decrease, McNemar mid-*p* = 0.021), while the increase of the frequency of strategies of negative resolution did not reach the level of significance (*p* = 0.375, McNemar mid-p). Conversely, the frequency of strategies of positive resolution increased in the resolution of internal conflicts and in the resolution of external conflicts (87% increase, McNemar mid-*p* = 0.020; and 78% increase, McNemar mid-*p* = 0.015, respectively), whereas the frequency of strategies involving negative resolution showed no changes in either situation (*p* = 0.500 for the negative resolution of internal conflicts and *p* = 0.480 for external conflicts; McNemar mid-p.). Finally, the frequency of both positive and negative strategies for the resolution of children’s setbacks increased in October compared with June (78% increase, *p* = 0.038; and 86% McNemar mid-*p* = 0.039, respectively).

**Fig. 5:**
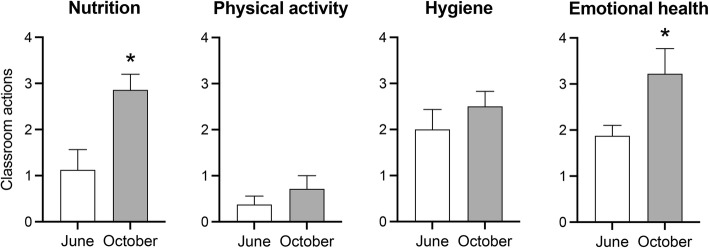
Effects of the training in observed classroom activities in the four all@once dimensions. Histograms show the number of classroom activities documented by pictures while teachers work in the classroom before (June) and after (October) training attendance. *, *p* < 0.05, paired Wilcoxon signed-rank test, different from June. (*n* = 16 teachers and 328 children per session)

The attendance of teachers at the all@once training also induced discrete changes in the number of activities engaged in by teachers and children in the classroom as captured by pictures. Some of these activities were related to the promotion of healthy habits through posters about healthy nutrition, such as promoting the intake of fruits and vegetables. Classroom activities also included the promotion of physical activity, such as playing games. Hand washing and classroom air circulation were the main hygiene promotion activities captured in pictures, while the promotion of emotional education was performed through posters expressing different emotions. Comparison of the number of documented classroom activities between June and October showed that the number of activities on healthy nutrition increased significantly in October (W = 21.00, *p* = 0.031). A similar increase in the number of classroom activities in emotional health was found (W = 28.00, *p* = 0.015), while no differences were observed in physical activity and hygiene activities (Fig. 5).

## Discussion

### Summary of the key findings of the study

Our pilot study aimed to cocreate, implement, and evaluate healthy habit promotion training for preschool teachers, with reference to the impact of such training on the teaching strategies used in the classroom with preschoolers. According to previous literature [50], cocreation design generates interventions that are more likely to have a positive impact on professional work. For this reason, researchers have highlighted the potential and evidence of cocreation as an effective strategy in the whole process of an educational intervention, including voices from all stakeholders, including children [63]. Our intervention sought to influence behavior and ensure applicability and context appropriateness, as assayed in previous studies that have focused on obesity management [64]. Our approach provided training organized into online capsules with a 360-degree holistic view of health in four main dimensions. Pilot testing of the all@once impact at the classroom evidenced an increased likelihood of observing healthy events from the four dimensions of the training, especially from the emotional health dimension.

Previous research has used multiple strategies to involve diverse voices in cocreation design, including workshops, observations, and interviews [65]. Discussion groups are an effective way of listening to what children have to say [66] by integrating their voices with those of teachers or family members [19, 59, 60]. Other studies have involved municipalities [40] or expert professionals [67]. We included all of these voices in our research, which also revealed the usefulness of the indirect observation of preschoolers and teachers as an effective strategy to assess classroom implementation. In this study, we also provide evidence that pictures are an applicable assessment technique [27].

Our pilot study integrated four dimensions to promote health during preschool through training for teachers following a holistic health concept. This clearly contrasts with previous studies that have involved only one dimension, such as nutrition [28, 42], physical activity [30, 37, 38, 68], hygiene [31], or emotional education [34]. Other studies have combined two of these elements, namely, nutrition and physical activity [69] or nutrition and emotional health [33].

Regarding our results in the nutrition dimension, the increased intake of fruits in our second assessment is noteworthy and in line with previous research [28]. Lunches were brought from home and could also be related to the likelihood of primary caregivers reviewing and modifying their practices [42] and thus to their inclusion in our cocreation process. In the physical activity and body awareness dimension, we linked our discrete results with the lack of space and equipment [38], which challenged teachers in promoting physical activity, although personal and structural barriers at both the individual teacher and broader systems levels could also be involved [37]. Finally, we should also consider that this dimension was relegated to lower levels of prioritization in the diamond technique during the cocreation sessions.

The all@once training showed little impact on the hygiene routines performed at the schools (e.g., in hand washing at different moments in school practice), which is in line with previous research [31]. However, our study reflects a positive effect of hand washing after children became dirty. We should keep in mind that all@once was implemented in 2019, before the global health emergency due to the coronavirus disease 2019 (COVID-19) pandemic made hand washing and air circulation mandatory [70]. Hygiene measures implemented for the COVID-19 pandemic may be an opportunity to form lifelong hand hygiene habits [71]. Even if they may lead to unintended consequences [72], hygiene habits at school have deeply improved, and the hygiene dimension of all@once training is thus of greater importance and interest.

In its holistic view of health, our training contemplated emotional health as a transversal dimension, according to the needs expressed by stakeholders in the cocreation sessions. This line of thought was followed in our analysis, paying special attention to teachers’ strategies when addressing this dimension. We found that teachers have an increased tendency to solve problems, such as internal and external conflicts and setbacks, which is in contrast with the statement that positive social relationships are valuable and foundational qualities for well-being beginning at the earliest ages and extending across all ages [73], although these issues were solved in a negative way, specifically regarding efforts to decrease attachment. Although only a minority of cocreation stakeholders were teachers participating in the impact assessment, we cannot discard the possibility of some bias regarding the fact that teachers showed a greater tendency to solve emotional-health-related problems. Nevertheless, the importance of promoting mental health among preschoolers and the importance of doing so in the middle and long term have been noted [34]. For this reason, researchers have highlighted the importance of promoting healthy emotional habits in the classroom. Furthermore, there is a connection between emotional health and overweight and obesity, which reinforces the idea of including this relationship in further training approaches [33].

Finally, the existing evidence suggests that a community-wide capacity-building approach to reducing child obesity is flexible, cost-effective, sustainable, equitable, and safe and has the potential to influence the underlying social and economic determinants of health [74]. These findings point to the personalized promotion of each of the all@once training dimensions to lead to better practices promoting health at school. This transversal promotion would be based on the complementarity of two models, namely, WSCC 5 and social and emotional learning (SEL) [75], as well as being in line with the 2030 SDGs [4].

Taking all of this into account, it is fair to mention the fact that changing behavior is a long-term issue. It is thus necessary to design longer-term interventions at a larger scale. Moreover, researchers’ presence in the classroom during ONPOs may influence the natural behavior of teachers and children. This reactivity bias could have influenced both the pretraining and posttraining ONPOs in the current study. To minimize this bias, we performed the observations at all times children were in school and not just at a specific moment of the day. Nevertheless, we would like to emphasize the utility of the pilot study in obtaining some significant changes in a short time period, with a small population participating in the assessment. This remains, therefore, an interesting line of research to pursue, as it involves more members of the educational community for longer periods.

### Limitations and strengths of the study

There are some limitations present regarding the design and development of this study. First, the observation sessions were conducted in different moments of the academic year (June and October), which could influence some variables, such as water intake and classroom air circulation. This also applies to the emotional health dimension, which could also be influenced by the different moments of the academic year in the attachment variable. Second, we detected differences in the use of the assessment instruments, despite training sessions to achieve systematic and homogeneous data collection from all observers. These differences influenced our data collection and analysis and should be taken into account in future research in the design and training stages. Third, to detect deeper changes in preschool teachers’ health strategies, an extension of training, implementation, and impact evaluation to the mid and long term is necessary. Moreover, this is a preliminary pilot study with a limited number of teachers and children that needs to be performed at larger performance scale to assess the all@once real impact on schools. Finally, we detected incongruities in some of the results from the cocreation sessions. For example, the idea of a school nurse was not raised in our program, despite all three sessions with teachers, families, and health professionals highlighting the importance of sanitary attention and first aid at schools; however, such a figure does not exist in Spain.

## Conclusions

In conclusion, using cocreation as a training design for teachers is productive in regard to promoting healthy habits for children, as it addresses the expressed needs of the community (teachers, families, and health professionals). Moreover, we found that the diamond technique for content prioritization is useful for integrating each voice into one. The resulting training, namely, all@once, presents a holistic approach to health (nutrition, physical activity, hygiene, and emotional health), which constitutes a novelty in the field. This approach effectively impacts routines at preschool and promotes healthy habits in the short term. Finally, an observational methodology that allows the inclusion of diverse contextual information that is collected in natural spaces of the schools and that involves qualitative data has been transformed and analyzed with quantitative analysis tools. These mixed method analyses appear to be useful approaches to assessing the impact of educational interventions on preventive health at schools. This may allow the analysis of future interventions involving other health dimensions that include human, material, ergonomic, and economic factors. This transversal integrative strategy outlines a promising path to continue expanding for inclusive education in integral health.

## Data Availability

The datasets generated and analyzed during the current study are not publicly available due to the need for informed consent for participation and bioethics agreement but are available from the corresponding author upon reasonable request. Files with anonymized data and templates will be available at the data repository from the Universitat de Barcelona (http://diposit.ub.edu/dspace/?locale=en).

## Abbreviations

CI: Confidence intervals
COVID-19: Coronavirus disease 2019
NO: Nonobserved
O: Observed
OR: Odds ratio
SDG: Sustainable development goals
SEL: Social and emotional learning
SEM: `Standard error of the mean
WSCC model: Whole school, whole community, whole child model
